# Governor vessel moxibustion for cancer-related fatigue in colorectal patients: a randomized trial

**DOI:** 10.3389/fonc.2023.1199200

**Published:** 2023-06-08

**Authors:** Huakang Li, Wei Huang, Kangming Du, Wei Liu, Ziliang Wu, Bo Xu, Qiang Li, Yue Wang, Bing Lin

**Affiliations:** ^1^ Department of Oncology, Hospital of Chengdu University of Traditional Chinese Medicine, Chengdu, Sichuan, China; ^2^ Department of Vascular Surgery, Hospital of Chengdu University of Traditional Chinese Medicine, Chengdu, Sichuan, China; ^3^ Department of Radiation Oncology, Sichuan Cancer Hospital, Chengdu, Sichuan, China; ^4^ Health Management Center, Hospital of Chengdu University of Traditional Chinese Medicine, Chengdu, Sichuan, China

**Keywords:** moxibustion, cancer-related fatigue, colorectal cancer, acupuncture, randomized controlled trial

## Abstract

**Objective:**

This study aimed to investigate the efficacy and mechanism of action of Governor Vessel Moxibustion (GVM) in the treatment of Cancer-Related Fatigue (CRF) in patients who have completed treatment for colorectal cancer.

**Methods:**

We randomly assigned 80 CRF patients in a 1:1 ratio to either the experimental group or the control group. During the three-week treatment period, both groups of patients received usual care for CRF provided by professional nurses. The experimental group received additional GVM treatment (three times a week, nine times total). The primary outcome was the mean change in total fatigue score from baseline to the end of treatment, assessed using the Chinese version of the Piper Fatigue Scale.

**Results:**

At baseline, the total fatigue scores were 6.20 ± 0.12 in the experimental group and 6.16 ± 0.14 in the control group. At the end of treatment, the total fatigue scores decreased by 2.03 points (32.7% decrease from baseline) in the experimental group and by 0.99 points (15.6% decrease from baseline) in the control group. The absolute reduction in total fatigue scores in the experimental group was 1.04 points higher than in the control group (95% CI, 0.93 to 1.15; *P*<0.001), corresponding to a relative difference of 17.1% (95% CI, 15.2% to 18.9%; *P*<0.001). At the end of treatment, the experimental group showed greater reductions in interleukin-6 (IL-6) and tumor necrosis factor-α (TNF-α) levels compared to the control group. No serious adverse events related to GVM treatment were observed.

**Conclusion:**

GVM appears to be safe and effective for alleviating CRF in patients who have completed colorectal cancer treatment, which may be related to the modulation of IL-6 and TNF-α levels.

**Trial registration:**

Chinese Clinical Trials Registry: ChiCTR2300069208.

## Introduction

Cancer-Related Fatigue (CRF) is one of the most common symptoms among cancer survivors, which is typically difficult to alleviate through adequate sleep or rest and significantly impairs quality of life (1). Cancer treatment is a major factor in the onset or exacerbation of CRF, including surgery, radiotherapy, chemotherapy, and biological therapy (2). The incidence of CRF during cancer treatment has been reported to range from 25% to 99%, with moderate to severe CRF occurring in 30% to 60% of cases (1). In colorectal cancer survivors, more than one-third continue to suffer from CRF for a year or longer after the end of cancer treatment (3). At present, there are no universally accepted and effective pharmacological treatments for CRF. Although interventions, including exercise therapy, cognitive-behavioral therapy, sleep management, and psychological counseling, can alleviate CRF, their effects are limited (4). Therefore, there is an urgent need to develop new strategies to alleviate the burdensome symptoms of CRF.

Moxibustion is a common alternative and complementary therapy. In traditional Chinese medicine, moxibustion and acupuncture share the same theoretical framework and treat diseases by stimulating acupoints. Unlike acupuncture that uses needles to stimulate acupoints, moxibustion applies heat stimulation to acupoints by burning herbal mugwort. Acupuncture has been used to treat CRF. A meta-analysis showed that acupuncture significantly alleviated CRF compared to sham acupuncture or usual care (5). Many studies suggest that moxibustion also has fatigue-improving effects. A meta-analysis showed that moxibustion effectively treated chronic fatigue syndrome (6). Several clinical studies on the effects of moxibustion on CRF have shown positive results, regardless of the type of moxibustion used (7, 8). Furthermore, compared to acupuncture, moxibustion has its unique advantage due to its thermal stimulation effect. Given that normal tissues and the tumor microenvironment exhibit different thermal sensitivities (9), moxibustion as a form of hyperthermia therapy can potentially exploit this distinction. Limited research has suggested that moxibustion can selectively disrupt colorectal cancer cells, while producing minimal damage to normal tissues (10).Therefore, moxibustion may be a promising strategy for managing CRF.

Governor Vessel Moxibustion (GVM) is a specific moxibustion treatment method. Compared to general moxibustion, GVM has the advantages of a wider treatment area, richer medicinal ingredients, and stronger penetration (11). Therefore, based on experience, we hypothesized that GVM could be an effective method for treating CRF. We conducted a pilot study, which showed initial feasibility and effectiveness of GVM for CRF treatment. Colorectal cancer patients were randomly assigned to either the usual care group or the GVM group, with 10 participants per group. The GVM group received GVM treatment three times per week for three weeks, in addition to receiving usual care. All patients completed the treatment with good compliance. At the end of treatment, the Piper Fatigue Scale total fatigue score decreased by 1.81 points from baseline in the GVM group (1.03 points in the usual care group). Moreover, inflammation is considered to play a key role in the pathogenesis of CRF (1), and moxibustion has been shown to reduce pro-inflammatory cytokine levels (12).

We hypothesized that GVM could alleviate CRF by modulating the inflammatory response. To further investigate the efficacy and underlying mechanisms of GVM in treating CRF, we conducted this randomized controlled trial.

## Methods

### Trial design

We conducted a single-center, open-label, parallel-group, assessor-blinded randomized controlled trial at Chengdu University of Traditional Chinese Medicine Affiliated Neijiang Hospital. 80 participants were randomly allocated in a 1:1 ratio to the experimental group and control group, with 40 patients in each group. The study duration was seven weeks, including a one-week screening period, a three-week treatment period, and a three-week follow-up period post-treatment. The study was approved by the hospital ethics committee and registered with the Chinese Clinical Trials Registry (ChiCTR2300069208). All patients provided written informed consent before randomization.

### Participants

Participants were recruited using a multimodal strategy, either referred by their clinical physicians or voluntarily participated in response to media advertisements. Inclusion criteria were as follows: meeting the International Classification of Diseases 10th Revision (ICD-10) diagnostic criteria for CRF; moderate to severe fatigue, defined as a Piper Fatigue Scale score of ≥4; histopathological diagnosis of colorectal cancer; stage I to III colorectal cancer (American Joint Committee on Cancer, 8th edition); completion of surgical treatment and necessary adjuvant chemotherapy; aged 18-80 years, with no gender restrictions; Karnofsky Performance Status (KPS) scores ≥70; and normal results for blood routine tests, liver and kidney function tests, and thyroid function tests. Exclusion criteria were as follows: pregnant or lactating women; psychiatric disorders or severe cognitive impairment; severe systemic diseases, such as cardiovascular diseases or acute infectious diseases; and use of medications for managing CRF within four weeks (dexamethasone, modafinil, burpropion, or methylphenidate).

### Interventions

During the three-week treatment period, both groups of patients received usual care for CRF administered by professional nurses. This care regimen included: 1) Exercise guidance: Patients were encouraged and monitored to engage in 180 to 300 minutes of moderate-intensity exercise per week, such as brisk walking, dancing, yoga, or Tai Chi; 2) Nutritional management: Using the Nutritional Risk Screening 2002 tool (13), a weekly nutritional risk screening was conducted and patients identified to be at nutritional risk were referred to the nutrition department for specialized treatment; 3) Sleep management: Patients were instructed to maintain regular daily routines, limit napping to no more than one hour, and use techniques such as foot soaking in warm water or massages to assist with sleep before bedtime; 4) Health education: Three weekly communication sessions were held with patients for approximately an hour each, during which essential knowledge on CRF management and tumor rehabilitation was covered, and psychological comfort was provided to help manage distressing emotions and enhance treatment confidence.

The experimental group received additional GVM treatment, which was administered three times a week, for a total of nine sessions. The GVM treatment area encompassed the spinal region from GV14 (Dazhui acupoint) to DU2 (Yaoyu acupoint) and extended to a three cm region on either side of the spine. The specific operational steps were as follows: 1) The patient was placed in the prone position, with the back fully exposed, and the GVM treatment area was disinfected three times with 75% alcohol; 2) Ginger juice was applied to the GVM treatment area, and it was covered with mulberry bark paper (12 cm wide, 70 cm long) aligned along the spine’s centerline; 3) A trapezoidal ginger paste (6 cm base width, 5 cm top width, and 3 cm height) was placed on the mulberry bark paper, extending from GV14 to DU2; 4) A cylindrical moxa cone (5 cm wide, 3 cm high) was placed on the ginger paste, with the length equal to the ginger paste; 5) The moxa cone was ignited and allowed to burn until extinguished. After extinguishing, the moxa cone was replaced twice more. The total time required to complete all steps was approximately two hours.

### Outcome measures

The primary outcome measure was the absolute change in the total score on the Piper Fatigue Scale from baseline to the end of the 3-week treatment period. Secondary outcome measures included the relative change (percentage change) in the total score on the Piper Fatigue Scale, as well as the absolute changes in both KPS scores and levels of inflammatory markers IL-6 and TNF-α.

The Piper Fatigue Scale is a well-validated scale for assessing the severity of fatigue in the cancer population (14). The scale consists of 22 numeric rating scales, assessing fatigue across four subdomains: behavioral (6 items), affective (5 items), sensory (5 items), and cognitive (6 items). Fatigue severity is described using a 10-point numeric scale: mild (1, 2), moderate (3–5), and severe (6–9). Assessments were made before treatment (baseline, T0), after 1 week of treatment (T1), after 2 weeks of treatment (T2), after 3 weeks of treatment (end of treatment, T3), and after 6 weeks of treatment (end of follow-up, T4).

The KPS scores was used to assess the patient’s physical functional status (15). The score range for this scale is 0-100, divided into increments of 10. Higher scores indicate better health status and quality of life. Serum levels of IL-6 and TNF-α were measured using enzyme-linked immunosorbent assay methods, with fasting blood samples collected from patients in the morning. KPS scores and inflammatory markers were evaluated at T0 and T3.

### Sample size

Based on the results of a pilot study, after 3 weeks of treatment, the total fatigue scores on the Piper Fatigue Scale for the experimental group and the control group were 4.37 ± 0.59 and 5.07 ± 0.73, respectively. Using PASS software (version 15.0), the calculated sample size was 35 patients per group (two-sided α = 0.05; 1-β = 0.99; 1:1 ratio). Subsequently, the sample size was adjusted to 40 patients per group based on an anticipated dropout rate of 10%.

### Randomization and blinding

An independent statistician used Stata software to generate a randomization sequence using a block randomization method (block size of 4, allocation ratio 1:1). The group allocation information was stored in sealed, opaque envelopes and kept by independent personnel. Patients received envelopes in the order of enrollment to obtain their group allocation.

Due to the unique nature of GVM treatment, achieving double-blinding was difficult. Therefore, this clinical trial was open-label, with both patients and physicians aware of group assignments. To minimize selection bias, outcome assessments were conducted by researchers blinded to group allocation, who were instructed not to discuss treatment to maintain blinding.

### Statistical analysis

Data were analyzed using SPSS software (version 26.0) based on the intention-to-treat principle. Missing values were imputed using the last observation carried forward method, except for the primary outcome analysis. The Shapiro-Wilk test was used to assess the normality of the continuous data. Data were presented as means and standard errors (continuous variables with a normal distribution), medians and interquartile ranges (continuous variables with a non-normal distribution), and frequencies and percentages (binary and categorical variables). To evaluate the longitudinal effects of the Piper Fatigue Scale scores over time, a generalized estimating equation model with an exchangeable correlation structure was used to analyze the differences in changes from baseline between groups at each time point. This statistical technique adjusts for the non-independence of observations over time. In this model, the change from baseline was treated as the dependent variable, with group, time, and group × time interaction as independent variables, and baseline values were adjusted (16). No imputation was performed for missing data, as this analysis model inherently considers the missing values issue, accommodating data missing due to incomplete assessments or dropout in randomized controlled trials (17). Between-group differences in changes from baseline in KPS scores were analyzed using the Mann-Whitney U test. Analysis of covariance was used to compare between-group differences in changes from baseline in IL-6 and TNF-α levels. In this model, the change from baseline was treated as the dependent variable, group as the independent variable, and baseline values as covariates (18). Eight pre-specified exploratory subgroup analyses were conducted for the primary outcome. Interaction test statistics were used to test the subgroup effects. Spearman correlation analysis was used to analyze the correlation between changes in Piper Fatigue Scale scores and changes in KPS scores and inflammatory markers. A significance level of 0.05 was assumed, and all significance tests were two-sided. This trial did not control for multiple hypothesis testing, and no adjustments were made to p-values and confidence intervals. Therefore, the results concerning secondary outcomes and subgroups should be interpreted as exploratory.

## Results

### Participants and baseline characteristics

**Figure 1:**
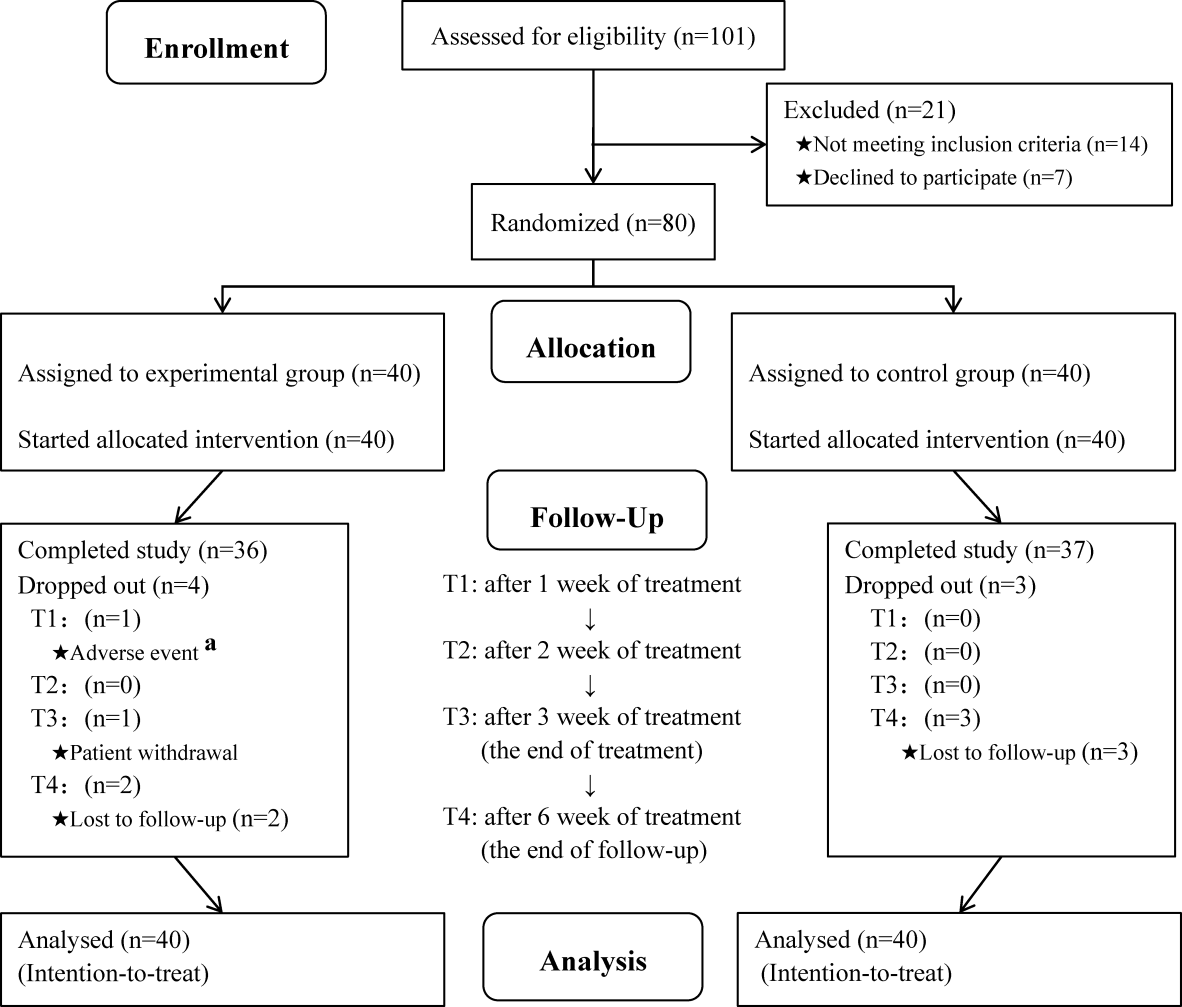
Flow of participants through each stage of the trial. ^a^although treatment was discontinued due to adverse events, this patient still continued to undergo visits and assessments.

Between March 2022 and February 2023, a total of 80 patients were recruited and randomly allocated to the experimental group (n=40) or control group (n=40). 36 patients in the experimental group and 37 patients in the control group completed the study (**Figure 1**). Of the seven patients who did not complete the study, one withdrew from the GVM treatment due to an allergic reaction, one requested to withdraw from the trial due to the novel coronavirus outbreak, and five were lost to follow-up. At the end of treatment and at follow-up, 79 and 74 patients respectively, provided complete data during their research visits. The baseline characteristics of the two groups were generally balanced (**Table 1**).

**Table 1:** Baseline characteristics of the participants.

Characteristic	Experimental group (n=40)	Control group (n=40)
Age, years, mean ± SE	59.70 ± 1.41	58.75 ± 1.42
Gender		
Male	13 (32.5%)	10 (25.0%)
Female	27 (67.5%)	30 (75.0%)
Cancer type		
Rectal cancer	12 (30.0%)	14 (35.0%)
Colon cancer	28 (70.0%)	26 (65.0%)
Cancer stage		
I	7 (17.5%)	6 (15.0%)
II	19 (47.5%)	24 (60.0%)
III	14 (35.0%)	10 (25.0%)
Adjuvant chemotherapy		
No	10 (25.0%)	8 (20.0%)
Yes	30 (75.0%)	32 (80.0%)
Chemotherapy protocol		
No chemotherapy	10 (25.0%)	8 (20.0%)
CAPEOX	7 (17.5%)	7 (17.5%)
FOLFOX	13 (32.5%)	11 (27.5%)
FOLFIRI	5 (12.5%)	6 (15.0%)
Other protocol	5 (12.5%)	8 (20.0%)
KPS score		
70	8 (20.0%)	7 (17.5%)
80	23 (57.5%)	27 (67.5%)
90	9 (22.5%)	6 (15.0%)
Fatigue level		
Moderate	16 (40.0%)	15 (37.5%)
Severe	24 (60.0%)	25 (62.5%)
TCM typing		
Cold	27 (67.5%)	29 (72.5%)
Heat	13 (32.5%)	11 (27.5%)

SE, standard error; CAPEOX, capecitabine and oxaliplatin; FOLFOX, folinic acid, 5-fluorouracil, and oxaliplatin; FOLFIRI, folinic acid, 5-fluorouracil, and irinotecan; KPS, Karnofsky Performance Status; TCM, Traditional Chinese Medicine.

### Primary outcome

**Figure 2:**
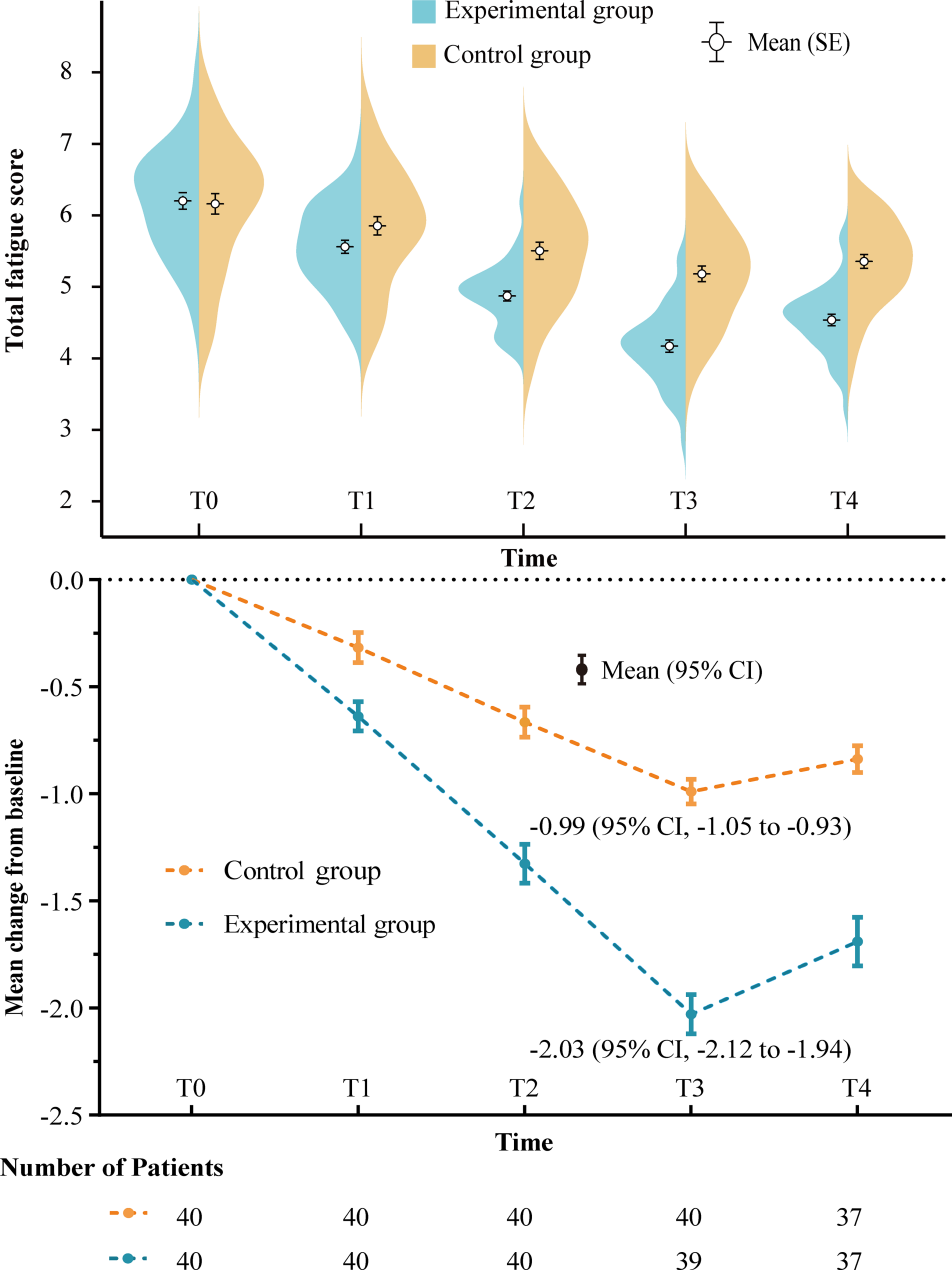
Change in total fatigue score over time. The curve depicts the density distribution. Numbers of patients at each time point are those with evaluable data per treatment group. SE, standard error; CI, confidence interval; T0, baseline; T1, after 1 week of treatment; T2,after 2 weeks of treatment; T3, after 3 weeks of treatment (end of treatment); T4, after 6 weeks of treatment (end of follow-up).

As shown in **Figure 2**, the total fatigue scores of both groups gradually decreased during treatment. However, the decrease in the experimental group was greater than that in the control group, and the difference between the two groups became more pronounced over time. Three weeks after the end of treatment, although the total fatigue scores of both groups rebounded, the inter-group differences remained significant. At baseline, the total fatigue scores of the experimental and control groups were 6.20 ± 0.12 and 6.16 ± 0.14, respectively. At the end of treatment, the total fatigue scores of the experimental and control groups decreased by 2.03 points (95% CI, 1.94 to 2.12) and 0.99 points (95% CI, 0.93 to 1.05), respectively. The decrease in the total fatigue score in the experimental group was 1.04 points (95% CI, 0.93 to 1.15; *P*<0.001) higher than that in the control group. Three weeks after the end of treatment, the decrease in the total fatigue score in the experimental group was still 0.85 points (95% CI, 0.72 to 0.98; *P*<0.001) higher than that in the control group. The changes in the fatigue scores of the four subdomains in both groups also showed similar trends (**Table 2**).

**Table 2:** Generalized Estimating Equation analysis for the comparison of total and subdomain fatigue scores.

Outcome	Mean ± SE		Adjusted^a^ mean change from baseline (95% CI)		Adjusted^a^ difference (95% CI)	*P* value (difference)
Time	Experimental	Control	Experimental	Control		
Total fatigue score						
T0	6.20 ± 0.12	6.16 ± 0.14	NA	NA	NA	NA
T1	5.56 ± 0.09	5.85 ± 0.13	-0.64 (-0.71to -0.57)	-0.32 (-0.39 to -0.25)	-0.32 (-0.42 to -0.22)	<0.001
T2	4.87 ± 0.07	5.51 ± 0.11	-1.33 (-1.42 to -1.23)	-0.67 (-0.74 to -0.59)	-0.66 (-0.78 to -0.55)	<0.001
T3	4.17 ± 0.09	5.18 ± 0.10	-2.03 (-2.12 to -1.94)	-0.99 (-1.05 to -0.93)	-1.04 (-1.15 to -0.93)	<0.001
T4	4.54 ± 0.08	5.36 ± 0.96	-1.69 (-1.80 to -1.58)	-0.84 (-0.90 to -0.77)	-0.85 (-0.98 to -0.72)	<0.001
Behavioral fatigue score						
T0	5.99 ± 0.15	6.18 ± 0.17	NA	NA	NA	NA
T1	5.33 ± 0.09	5.81 ± 0.14	-0.71 (-0.84 to -0.58)	-0.32 (-0.46 to -0.18)	-0.39 (-0.58 to -0.19)	<0.001
T2	4.62 ± 0.09	5.44 ± 0.12	-1.43 (-1.59 to -1.26)	-0.69 (-0.82 to -0.56)	-0.74 (-0.95 to -0.53)	<0.001
T3	3.78 ± 0.08	5.04 ± 0.10	-2.27 (-2.37 to -2.17)	-1.09 (-1.20 to -0.98)	-1.18 (-1.33 to -1.03)	<0.001
T4	4.14 ± 0.07	5.23 ± 0.08	-1.92 (-2.07 to -1.77)	-0.92 (-1.03 to -0.81)	-1.00 (-1.19 to -0.81)	<0.001
Affective fatigue score						
T0	6.29 ± 0.15	5.95 ± 0.17	NA	NA	NA	NA
T1	5.72 ± 0.14	5.70 ± 0.18	-0.54 (-0.68 to -0.39)	-0.30 (-0.42 to -0.19)	-0.23 (-0.42 to -0.05)	0.012
T2	5.16 ± 0.10	5.42 ± 0.16	-1.09 (-1.22 to -0.96)	-0.58 (-0.70 to -0.46)	-0.51 (-0.69 to -0.34)	<0.001
T3	4.57 ± 0.11	5.17 ± 0.17	-1.69 (-1.86 to -1.52)	-0.83 (-0.98 to -0.68)	-0.86 (-1.09 to -0.63)	<0.001
T4	4.95 ± 0.14	5.41 ± 0.13	-1.36 (-1.56 to -1.16)	-0.63 (-0.78 to -0.49)	-0.72 (-0.97 to -0.48)	<0.001
Sensory fatigue score						
T0	6.25 ± 0.15	6.26 ± 0.15	NA	NA	NA	NA
T1	5.53 ± 0.12	5.89 ± 0.15	-0.72 (-0.85 to -0.59)	-0.37 (-0.49 to -0.24)	-0.35 (-0.53 to -0.17)	<0.001
T2	4.79 ± 0.11	5.49 ± 0.14	-1.46 (-1.64 to -1.28)	-0.77 (-0.92 to -0.62)	-0.69 (-0.93 to -0.46)	<0.001
T3	4.14 ± 0.11	5.16 ± 0.14	-2.14 (-2.33 to -1.95)	-1.10 (-1.22 to -0.97)	-1.05 (-1.28 to -0.82)	<0.001
T4	4.44 ± 0.12	5.32 ± 0.13	-1.89 (-2.10 to -1.68)	-0.91 (-1.06 to -0.77)	-0.97 (-1.23 to -0.71)	<0.001
Cognitive fatigue score						
T0	6.31 ± 0.18	6.25 ± 0.19	NA	NA	NA	NA
T1	5.68 ± 0.17	6.00 ± 0.17	-0.62 (-0.72 to -0.52)	-0.26 (-0.32 to -0.19)	-0.36 (-0.48 to -0.24)	<0.001
T2	4.96 ± 0.12	5.65 ± 0.16	-1.34 (-1.48 to -1.21)	-0.60 (-0.70 to -0.51)	-0.74 (-0.91 to -0.58)	<0.001
T3	4.32 ± 0.14	5.29 ± 0.16	-1.95 (-2.08 to -1.82)	-0.97 (-1.07 to -0.87)	-0.98 (-1.15 to -0.81)	<0.001
T4	4.67 ± 0.12	5.47 ± 0.17	-1.63 (-1.78 to -1.47)	-0.84 (-0.97 to -0.71)	-0.79 (-0.99 to -0.59)	<0.001

a Regression models are adjusted for baseline value. SE, standard error; T0, baseline; T1, after 1 week of treatment; T2, after 2 weeks of treatment; T3, after 3 weeks of treatment (end of treatment); T4, after 6 weeks of treatment (end of follow-up).

### Secondary outcomes

At the end of treatment, the mean percent change from baseline in total fatigue scores demonstrated a decrease of 32.7% (95% CI, 31.3% to 34.2%) in the experimental group and 15.6% (95% CI, 14.5% to 16.8%) in the control group, respectively. The decrease in the experimental group was greater than that in the control group (difference, 17.1%; 95% CI, 15.2% to 18.9%; *P*<0.001).

At baseline, the KPS scores for both groups were 80 (80, 80) points. At the end of treatment, the experimental and control groups had scores of 90 (80, 90) and 80 (80, 90) points, respectively. Compared to baseline, the experimental and control groups improved by 5 (0, 10) and 0 (0, 10) points, respectively, with a statistically significant inter-group difference (Z=-1.989, *P*=0.047).

At the end of treatment, the IL-6 and TNF-α levels in the experimental group decreased by 4.27 pg/mL and 14.05 pg/mL compared to baseline, respectively. In contrast, the control group showed decreases of 1.75 pg/mL and 6.02 pg/mL, respectively. The experimental group exhibited greater decreases in IL-6 and TNF-α levels than the control group, with differences of 2.52 pg/mL (95% CI, 2.10 to 2.94; *P*<0.001) and 8.03 pg/mL (95% CI, 6.90 to 9.16; *P*<0.001), respectively (**Table 3**).

**Table 3:** Changes in IL-6 and TNF-α levels over time.

Outcome	Group	Mean ± SE		Adjusted ^a^ mean change from baseline (95% CI)	Adjusted ^a^ difference (95% CI)	*P* value (difference)
		T0	T3			
IL-6 (pg/mL)	Experimental	14.24 ± 0.33	9.85 ± 0.32	-4.37 (-4.62 to -4.12)	-2.62 (-2.97 to -2.27)	<0.001
	Control	13.85 ± 0.31	12.13 ± 0.30	-1.75 (-1.99 to -1.50)		
TNF-α (pg/mL)	Experimental	42.07 ± 1.01	28.10 ± 0.97	-14.47 (-15.08 to -13.86)	-8.46 (-9.32 to -7.59)	<0.001
	Control	43.42 ± 1.14	37.33 ± 1.10	-6.01 (-6.62 to -5.40)		

a Regression models are adjusted for baseline value. SE, standard error; T0, baseline; T3, after 3 weeks of treatment (end of treatment); IL-6, interleukin-6; TNF-α,tumor necrosis factor-α.

### Subgroup analysis

**Figure 3:**
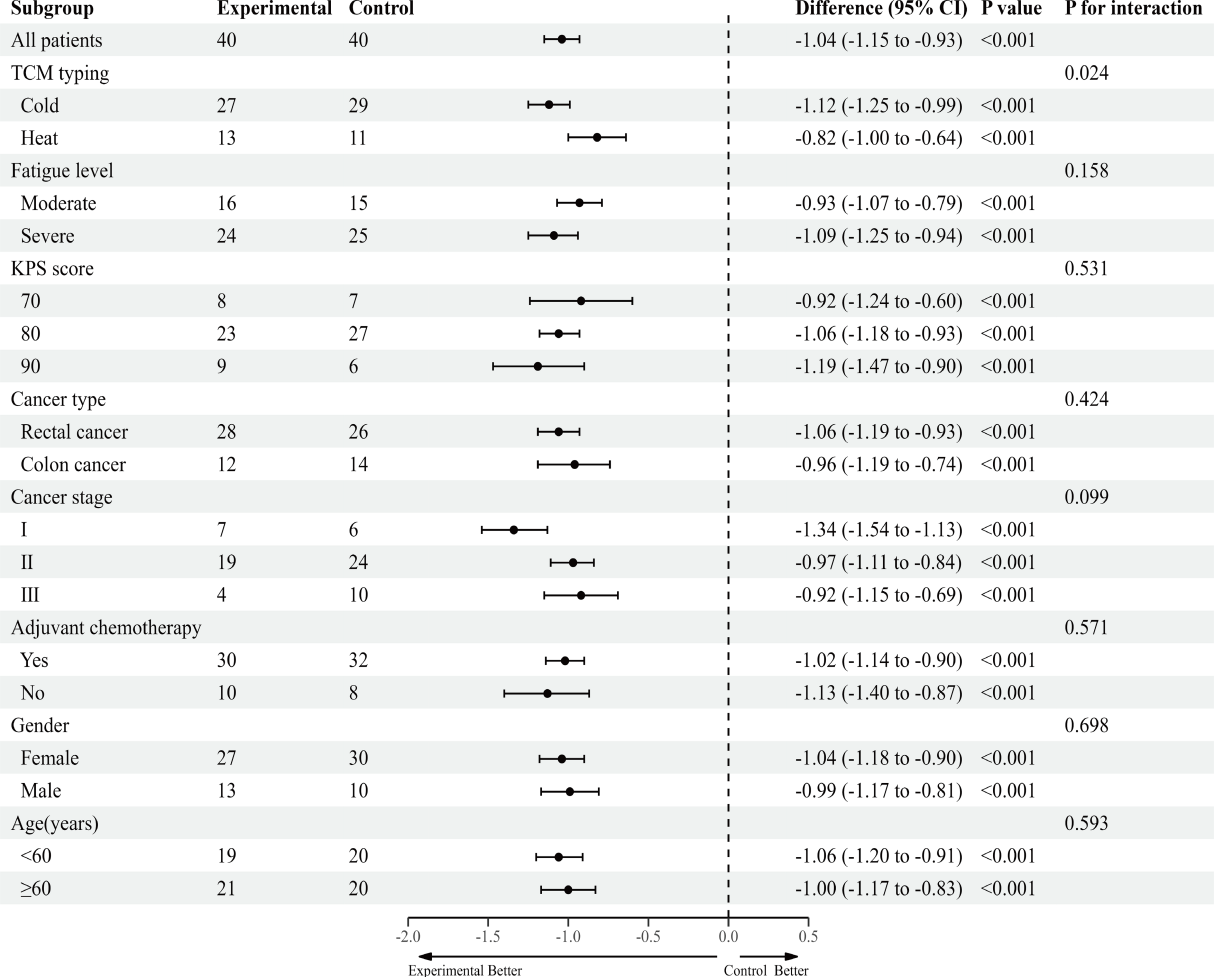
Exploratory subgroup analysis of primary outcome, according to participant characteristics at baseline. TCM, Traditional Chinese Medicine; KPS, Karnofsky Performance Status.

Consistent with the overall results, point estimates in all predefined subgroups showed a superior treatment effect of CRF in the experimental group compared to the control group (**Figure 3**). Notably, a statistically significant interaction was observed between the Traditional Chinese Medicine (TCM) typing subgroup and the treatment group (*p* for interaction effect estimate=0.024). This suggests that compared to the heat type CRF, the cold type CRF demonstrates a more sensitive trend toward GVM treatment.

### Correlation analysis

At the end of treatment, the change in total fatigue score from baseline was significantly positively correlated with changes in IL-6 and TNF-α (r=0.73 and r=0.71, *p*<0.001 for both), and negatively correlated with changes in KPS score (r=-0.27, *p*=0.016).

### Safety analysis

Mild adverse events related to GVM treatment occurred in 10.0% (4/40) of the patients in the experimental group. One patient experienced a rash with itching in the treatment area during the second GVM session, which improved after symptomatic treatment. Three patients developed local erythema, which resolved spontaneously within one week after the end of treatment.

## Discussion

The risk of CRF in colorectal and breast cancer is higher than in other types of cancer (19). Previous clinical trials on CRF have mostly focused on breast cancer patients (20), with fewer studies targeting colorectal cancer patients. Our randomized controlled trial demonstrated that a three-week course of GVM treatment could effectively ameliorate CRF in patients who had completed treatment for colorectal cancer. Moreover, the therapeutic effect persisted for at least three weeks following the conclusion of the treatment. Regarding safety, the incidence of adverse reactions to GVM was 10.0%, but these reactions were mild and transient. Importantly, they did not adversely impact the patients’ quality of life. Furthermore, compliance with the GVM treatment regimen was exceptionally high, with 95.0% (38/40) of patients completing the treatment.

Our study also showed an increase in KPS scores for patients after GVM treatment, signifying an improvement in their quality of life. Correlation analysis revealed that the increase in KPS scores was associated with a decrease in total fatigue scores, indicating that GVM may improve quality of life by alleviating CRF. Furthermore, GVM may be an approach to manage clusters of symptoms rather than only single symptoms. Positive trial results have also been observed for GVM in improving pain, insomnia, depression, nausea, vomiting, diarrhea, anorexia, and myelosuppression (11), suggesting that GVM may improve quality of life in multiple aspects.

TCM typically classifies CRF into cold and heat types. In our study of 80 CRF patients, the proportion of cold type was higher than heat type (7:3), consistent with previous reports (7, 21). According to TCM theory, GVM can promote circulation in the meridian system, exerting a bidirectional regulatory effect that is applicable to both cold and heat types of CRF. Moreover, because of its thermal stimulation properties, GVM might exhibit a more pronounced therapeutic effect on the cold type CRF. The outcomes of our subgroup analysis aligned with the predictions of TCM theory, providing some scientific evidence for TCM classification of CRF. Certainly, since the results of the subgroup analysis are exploratory, further research is needed to validate the guidance of TCM typing for GVM treatment of CRF.

Various biological mechanisms of CRF have been proposed and studied (1, 4). These include inflammation, hypothalamic-pituitary-adrenal axis dysfunction, serotonin neurotransmitter imbalance, and mitochondrial dysfunction. Among them, inflammation has received the most empirical attention and support (22). Moxibustion is thought to have immunomodulatory effects (23). Due to funding limitations, we selected only IL-6 and TNF-α among the numerous inflammatory markers in this study, as they have the most evidence for their association with fatigue and moxibustion (24, 25). Our study results further suggested that the anti-inflammatory effect might be one of the potential mechanisms of GVM treatment for CRF. Given the promising clinical effects observed in the current study, further research is needed to elucidate the therapeutic mechanisms of GVM for CRF.

Our trial possesses several noteworthy limitations. Firstly, our study was a single-center phase 2 clinical trial with a limited sample size. To validate the results of our study, larger multi-center clinical trials are needed. Secondly, our trial population included only patients who had completed cancer treatment, excluding those who underwent surgery, radiotherapy, or chemotherapy during the study period. Therefore, the effectiveness and safety of GVM for CRF before or during cancer treatment need further investigation. Thirdly, the follow-up period was only 3 weeks, and longer-term studies are needed to explore the sustained effects of GVM treatment for CRF. Lastly, our trial only evaluated the efficacy of GVM for CRF. Future research should consider integrating diverse moxibustion methodologies to probe the optimal moxibustion technique for managing CRF.

## Conclusion

In conclusion, GVM may be a safe and effective approach to alleviate CRF in patients who have completed colorectal cancer treatment. Its therapeutic effects may be related to the modulation of IL-6 and TNF-α levels. Furthermore, future research could investigate the potential of GVM in different cancer types, explore the combination of GVM with pharmacological treatments, and evaluate the long-term effects of GVM for CRF. Such efforts could contribute to a more comprehensive understanding of GVM’s clinical applications and potentially enhance its therapeutic effects.

## Data availability statement

The original contributions presented in the study are included in the article/supplementary material. Further inquiries can be directed to the corresponding author.

## Ethics statement

The studies involving human participants were reviewed and approved by Chengdu University of Traditional Chinese Medicine Affiliated Neijiang Hospital. The patients/participants provided their written informed consent to participate in this study.
